# Maxillofacial prosthodontics practice profile: a survey of non-United States prosthodontists

**DOI:** 10.1186/s40463-017-0211-5

**Published:** 2017-04-27

**Authors:** Nina Ariani, Harry Reintsema, Kathleen Ward, Cortino Sukotjo, Alvin G. Wee

**Affiliations:** 1 Department of Prosthodontics, Faculty of Dentistry, Universitas Indonesia, Jakarta, Indonesia; 2 Division of Preventive Dentistry, Niigata University Graduate School of Medical and Dental Sciences, Niigata, Japan; 3 Department of Oral and Maxillofacial Surgery, University of Groningen, University Medical Center Groningen, Groningen, The Netherlands; 4 Creighton University School of Dentistry, Omaha, NE USA; 5 Department of Restorative Dentistry, University of Illinois at Chicago, College of Dentistry, Chicago, IL USA; 6 Department of Prosthodontics, Creighton University School of Dentistry, Omaha, NE USA; 7 Department of Surgery, Dental Service, Veterans Affairs Nebraska-Western Iowa Healthcare System, 4101 Woolworth Ave, Omaha, NE 68105 USA

**Keywords:** Prosthodontics, Oral oncology, Rehabilitation, Career decision

## Abstract

**Background:**

This study surveyed non-United States maxillofacial prosthodontists (MFP) to determine their practice profile and rationale for pursuing an MFP career.

**Methods:**

Email addresses for the MFP were obtained from the International Society for Maxillofacial Rehabilitation, American Academy of Maxillofacial Prosthetics, and International Academy for Oral Facial Rehabilitation. Emails with a link to the electronic survey program were sent to each participant. Chi-square and Mann–Whitney-U tests were used to investigate the influence of formal MFP training on professional activities and type of treatments provided.

**Results:**

One hundred twelve respondents (response rate 39%) from 33 nationalities returned the survey. The top three reasons for pursuing an MFP career were personal satisfaction, prosthodontics residency exposure, and mentorship. The predominant employment setting was affiliation with a university (77%). There were significant differences between respondents with and without formal MFP training regarding provision of surgical treatments (*P* = 0.021) and dental oncology (*P* = 0.017). Most treatments were done together with otolaryngology, oral surgery (68%) and head and neck surgery (61%). Practitioners not affiliated with a university spent significantly more time in clinical practice (*P* = 0.002), whereas respondents affiliated with universities spent significantly more time in teaching/training (*P* = 0.008) and funded research (*P* = 0.015).

**Conclusions:**

Personal satisfaction is the most important factor in a decision to choose an MFP career. Most of the MFPs work at a university and within a multidisciplinary setting. There were differences regarding type of treatments provided by respondents with and without formal MFP training.

## Background

Maxillofacial prosthetics is a subspecialty of prosthodontics that deals with rehabilitation of patients with defects or disabilities caused by trauma, tumor, or congenital disorders [1]. Prostheses are made to replace teeth, lost bone, or soft tissue to restore oral function and esthetics. Prosthetic devices also are fabricated to shield and protect the facial structure during radiotherapy. Sometime, facial or body prosthesis is fabricated for psychosocial reasons. Given the vast services provided to patients as illustrated above, maxillofacial prosthodontists (MFP) are trained to work in a multidisciplinary setting together with oral surgeons; ear, nose, and throat surgeons; plastic surgeons; speech pathologists; etc. [2].

There are a number of professional organizations dealing with maxillofacial prosthodontics. The mission of the International Society for Maxillofacial Rehabilitation (ISMR) is to advance interdisciplinary maxillofacial rehabilitation through education, patient care, outreach and research. The International Academy for Oral Facial Rehabilitation (IAOFR) is a small international group of surgeons and prosthodontists (fewer than 50 fellows) with particular interest in optimizing treatment outcomes of surgical-prosthetic interventions. In the United States, the American Academy of Maxillofacial Prosthetics (AAMP) is an association of prosthodontists who are engaged in the art and science of maxillofacial prosthetics. The academy has approximately 300 fellows devoted to the study and practice of methods used to habilitate the esthetics and function of patients with acquired, congenital, and developmental defects of the head and neck. Methods used to maintain the oral health of patients exposed to cancercidal doses of radiation or cytotoxic drugs is also of interest to this association.

In the United States, training for prosthodontists in this area of maxillofacial prosthetic rehabilitation is unique in that most who are interested in this area have one year of additional training in maxillofacial prosthodontics, which is recognized by the Commission on Dental Accreditation [3, 4]. In a survey of dental schools worldwide, an average of 62 h with a range of 10–200 h were provided for maxillofacial prosthodontics instruction at the graduate level. However, 7% of dental schools presented the topics as lectures only, 62% had courses with lectures and clinical and laboratory exposure, while 28% had lecture/clinical courses with no laboratory component [5]. Training and recognition of MFP is not uniform globally.

To our knowledge, there is no published information regarding the number of MFP providing their services to society. Furthermore, there is only one known publication on the practice of MFPs in the UK, but this study is limited to maxillofacial technicians’ practice of silicone maxillofacial prostheses [1]. No published data is available on the characteristics of the practice of MFPs, such as what services individuals trained in maxillofacial prosthodontics provide to their patients, whether they are practicing their specialty, and how much they practice compared to general prosthodontics. Since US and non-US training is different for maxillofacial prosthodontics, this study focuses on the non-US practice profile. This information, along with background information, such as why they enter the specialty, is investigated in this study to understand more about trends in maxillofacial prosthodontic practices outside the United States. The aim was to map the availability of these services for patients and reveal the need for educational facilities around the globe.

The purpose of the study was to characterize non-US MFP practice profiles and their rationale for the decision to pursue maxillofacial prosthodontics training.

## Methods

### Questionnaire

A 28-items questionnaire was developed specifically for this study. The first part covered personal information: gender, age, country, affiliation, salary, and professional background. The second part covered the decision to pursue a career in MFP. The third part consisted of questions about maxillofacial prosthodontics treatment provided and multidisciplinary care.

The MFPs’ email addresses were obtained from the 2014 membership directories of ISMR, AAMP, and IAOFR. Since dentists, prosthodontists and anaplastologists members provide MFP services as well (e.g. specifically facial prostheses), they were included in the survey. An email with a link to the BlueQ electronic survey program from Creighton University was sent to each participant. The survey delivery protocol followed the Dillman Total Design Survey methodology [6]. A total of four emails were sent to the respondents. One week after the first email, the same email was sent again. Three and seven weeks from the initial email, the email containing the survey link was again sent only to participants who had not responded.

### Analysis

The data on the questionnaires were entered into an Excel spreadsheet (Microsoft Corporation, Redmond, WA). Blank or unclear responses were considered as missing. Descriptive statistics were given as percentages (%). The data was not normally distributed, and thus Chi-square and Mann–Whitney-U Tests (SPSS IBM, New York, NY) were used to investigate the influence of formal maxillofacial prosthodontics training on professional activities and type of treatments provided. One-way repeated measures ANOVA was considered a robust test against the normality assumption and therefore used for the most important factors for pursuing maxillofacial prosthodontics career.

## Results

### Response rate

Surveys were initially sent out to a sub total of 316 potential individuals, including to anaplastologists as they are part of workforce that provides MFP services. Forty entries were eliminated from the 316 potential individuals, resulting in 276 individuals who were surveyed. Of the 40 that were eliminated, 28 were duplicates, nine could not be contacted as their emails bounced, one was US maxillofacial prosthodontist and two were physical therapists. Total number of responses was 115 for a response rate of 41.6% (115/276). Five individuals who were not DDS, prosthodontists nor anaplastologists and three responses that provide no answers at all were excluded for a 39% response rate (107/276).

Respondents could provide information as they wished and were not required to complete all items. Therefore, the sample size of individual items varies. Non-response was regarded as missing.

### Demographic data

Thirty-three nationalities working in 32 different countries across five continents participated in the study: Australia (*n* = 8), Africa (*n* = 4), America excluding USA (*n* = 20), Asia (*n* = 37) and Europe (*n* = 38). The following are the countries where the participants work: Australia, Brazil, Canada, Chile, Colombia, Cyprus, Egypt, France, Germany, Greece, India, Iraq, Israel, Italy, Japan, Kenya, Libya, Malaysia, Mexico, Nepal, Netherlands, New Zealand, Peru, Saudi Arabia, Serbia, Singapore, South Africa, Spain, Sweden, Switzerland, Turkey, and United Kingdom. The three countries with the highest percentage of respondents were India (18, 17%), the Netherlands (15, 14%), and Canada (10, 9%). The majority of respondents were male (77%). The age of the respondents ranged from 26–71 years old (mean = 46 ± 11).

Respondents were asked to provide their education level and allowed to mark all that apply. Seventy-four responded with DDS/DMD/BDS, 62 held Master degree, 31 respondents were PhD and 35 respondents had certifications such as prosthodontics and fellow in MFP.

### Maxillofacial training and professional organizations

Seventy-one percent of respondents had formal maxillofacial prosthetics training. The training could either be part of or separate from prosthodontics specialty training. Eleven percent of those with formal maxillofacial training stated they did the training in an institution in the United States or Canada. They were not required to specify from which of those two countries they graduated.

When asked about professional background, 81% responded with maxillofacial prosthodontist, 2% were anaplastologists, 3% were prosthodontists, 1% were oral surgeons with formal maxillofacial prosthodontics training, 13% were other. The 13% who responded with ‘other’ stated they received formal MFP training or having at least a DDS/DMD/BDS degree and attend to patients. Thirty-two percent learned maxillofacial prosthodontics from colleagues, 30% from continuing education, and 38% either from an undergraduate/graduate program, observation program, the internet, textbooks, seminars, or experience.

A one-way repeated measures ANOVA was conducted to compare factors deemed important for an individual’s decision to pursue a career in maxillofacial prosthodontics. The results showed a statistically significant difference in the various factors (*p* <0.0001). Multiple pairwise comparisons were carried out between factors, with adjusted α = 0.05/45 = 0.001 after Bonferroni correction for multiple comparisons. Table 1 reveals the means (standard deviation) and also which factors are significantly different from each other. There were differences in scores across the different factors (*P* < 0.05). Personal satisfaction was the most important factor for the decision.

**Table 1:**
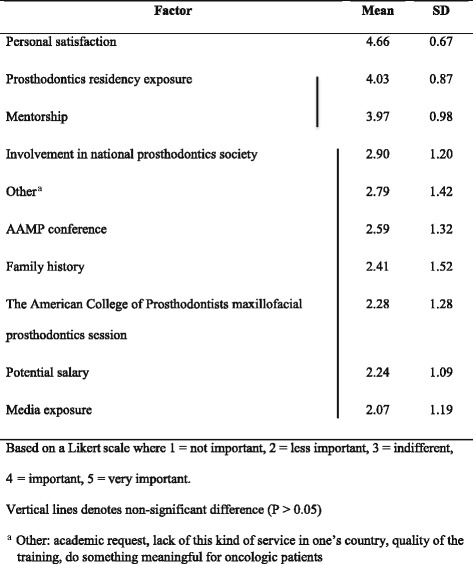
Factors important for decision to pursue maxillofacial prosthodontics career (*N* = 29)

Training facilities in the various countries were rated rather low by respondents, with 51% unsatisfied, 43% satisfied, and 6% very satisfied with training facilities available in their country. However, only 23.7% of respondents were not satisfied with their own maxillofacial prosthodontics training. For the remaining respondents, 44.7% were satisfied and 31.6% were very satisfied with their training.

There were no statistically significant differences between respondents with or without formal maxillofacial prosthodontics training regarding academic ranking at school (*P* = 0.101), salary (*P* = 0.103), involvement in national prosthodontics organizations (*P* = 0.713) and national MFP organizations (*P* = 0.516), and satisfaction working as maxillofacial prosthodontics (*P* = 0.636) (Table 2).

**Table 2:** Profiles of practitioners with vs without formal MFP training

	Formal MFP training (%)	Without formal MFP training (%)	P
Academic rank			
Top 5%	50	27	0.101
6–10%	11	27	
11–25%	0	18	
26–50%	14	9	
Not known	25	18	
Salary (in USD)			
≤ $ 50.000	24	7	0.103
$ 50.001–100.000	28	38	
$100.001–200.000	25	17	
≥ $200.001	23	38	
Fellow/member of national prosthodontics organizations			
Yes	82	83	0.713
No	7	10	
Organization does not exist	11	7	
Fellow/member of national MFP organizations			
Yes	38	31	0.516
No	7	14	
Organization does not exist	55	55	
Satisfaction working as MFP			
Unsatisfied	14	28	0.636
Satisfied	32	28	
Very satisfied	54	46	

### Maxillofacial prosthodontics practice and multidisciplinary care

**Fig. 1:**
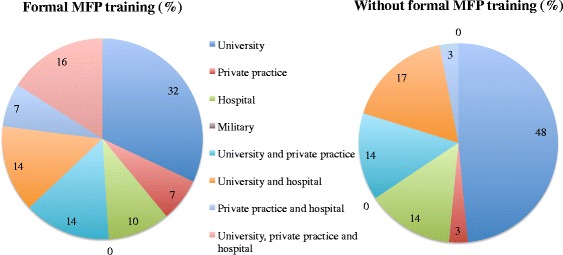
Affiliations of practicing respondents

There were no statistically significant difference in affiliations (Fig. 1) between those with formal and non-formal training (*P* = 0.302). The predominant employment setting for respondents with formal and non-formal training was related to universities (77%).

The percentage of respondents providing various types of treatment is presented in Table 3. Included in standard prosthodontics treatment are complete dentures, removable partial dentures, fixed partial dentures, and restoring implants. There were significant differences between respondents with and without formal MFP training regarding provision of surgical treatments (*P* = 0.021) and dental oncology (*P* = 0.017).

**Table 3:** Types of treatment provided

	Formal MFP training (%)		Without formal MFP training (%)		P
	Mean	Median	Mean	Median	
Types of treatment provided					
•Standard prosthodontics treatment	34 ± 33.6	30	39 ± 33.8	40	0.672
•General dentistry	22 ± 25.9	10	21 ± 22.7	11	0.828
•Maxillofacial prosthodontics	36 ± 27.8	23	37 ± 27.9	38	0.815
•Surgical	8 ± 11.0	5	3 ± 9.5	0	0.021*
Types of maxillofacial treatment provided					
•Trauma	8 ± 8.6	5	4 ± 5.2	0	0.182
•Mandibular resection	12 ± 11.3	10	22 ± 14.9	28	0.068
•Obturation	26 ± 17.5	20	38 ± 26.0	40	0.268
•Maxillofacial implant cases	12 ± 15.8	7.5	7 ± 12.5	0	0.115
•Facial prosthetics	13 ± 17.9	5	6 ± 8.3	0	0.169
•Dental oncology	12 ± 14.1	7.5	2 ± 4.2	0	0.017*
•Speech aid	3 ± 4.5	1	2 ± 3.4	0	0.144
•Palatal drop	2 ± 2.7	0	1 ± 1.6	0	0.079
•Palatal lift	3 ± 4.6	0	2 ± 3.4	0	0.227
•Naso-alveolar moulding	1 ± 3.3	0	1 ± 3.0	0	0.477
•Prosthetic treatment of cleft	5 ± 5.4	5	7 ± 9.5	0	0.862
•Radiation intra oral devices	4 ± 10.0	0	1 ± 1.6	0	0.068

*Statistically significant difference (*P* ≤ 0.05)

The multidisciplinary nature of MFP treatment is reflected in Table 4. The respondents were asked to indicate all disciplines with which they work when treating patients. Most treatments were done in conjunction with otolaryngology (68%) and oral surgery (68%) followed by head and neck surgery (61%).

**Table 4:** Percentage of respondents practising multidisciplinary treatment with other disciplines (*N* = 107)

Disciplines	Percent
No multidisciplinary treatment	5
Otolaryngology	68
Head and Neck Surgery	61
Oral Surgery	68
Plastic surgery	45
Oral Pathology / Oral Medicine	40
Radiation Oncology	50
Medical Oncology	26
Sleep Medicine	16
Other disciplines: psychiatry, dermatology, rheumatology, nephrology, speech therapy, neurosurgery	18

There were significant differences between practitioners affiliated and not affiliated with a university regarding percentage of time for professional activities (Table 5). Practitioners not affiliated with a university spent a significantly higher percentage of time in clinical practice (*P* = 0.002), whereas respondents affiliated with universities spent significantly more time in teaching/training (*P* = 0.008) and working on funded research (*P* = 0.015).

**Table 5:** Time for professional activities

	Affiliated to university		Not affiliated to university		P
	Mean	Median	Mean	Median	
Hours/week seeing patients	29 ± 14.9	30	31 ± 14.8	40	0.251
Percentage of time for professional activities					
Clinical practice	44 ± 23.4	40	69 ± 14.6	70	0.002*
Teaching or training	26 ± 17.7	30	9 ± 7.3	10	0.008*
Funded research	6 ± 10	0	0 ± 0	0	0.015*
Non-funded research	7 ± 8	5	3 ± 6.0	0	0.192
Supervision of personnel	5 ± 4.6	5	6 ± 11.8	0	0488
Non-clinical administration work	9 ± 9.1	10	12 ± 11.1	10	0.347

*Statistically significant difference (*P* ≤ 0.05)

There was no statistically significant difference in the satisfaction of working as maxillofacial prosthodontists (*P* = 0.636) between respondents with and without formal maxillofacial prosthodontics training. Ninety-eight percent would recommend the MFP profession to other colleagues.

## Discussion

The response rate of this study was 39%. There have been some variations in the response rates of surveys of maxillofacial prosthodontists. In 1992, a survey among members of the AAMP, ACP, and American Anaplastology Association had a response rate of 26% [7]. A survey in 2010 yielded a usable response rate of 22% [1], and another survey on MFPs reported a 16% response rate [8]. The response rate of this study gave a reasonable sampling for this population; future studies should include strategies to improve MFP response rates [9].

MFPs from 32 countries participated in this study. The majority of the respondents were male, indicating that either MFP is more popular among males or more males are active in the professional organization, as the respondents of this study were from the ISMR, AAMP, and IAOFR 2014 membership directories. It is also possible that a higher percentage of females chose not to respond to the survey.

Despite 51% of the respondents being unsatisfied with the training facilities in their home countries, when asked further about their own training 77% of respondents were either satisfied or very satisfied regardless of the quality of the training facilities. Eleven percent of respondents completed MFP training in the United States or Canada institution. There are also some prominent MFP centers available in Asia, Europe, North America, and South Africa that providing MFP trainings [10, 11]. The decision to pursue training abroad at an institution with better training facilities may be the cause of the MFP training satisfaction.

The most important factor in the decision to pursue MFP as a career is personal satisfaction, followed by prosthodontics residency exposure. Net earnings have been described as one of major determinants of choosing advanced education [12]. However, the potential salary ranked low as a factor important for the decision to pursue MFP career. Mentoring, interest among dental students, literature concerning the need for the profession in the future, and marketing of the profession as a career are identified as factors impacting the increase in the applicant pool for prosthodontics training [13]. These could fit into the MFP profession as well. Putting emphasis on prosthodontics residency exposure might ignite interest in the profession and help overcome this problem, since prosthodontics training is more readily available around the globe. Prosthodontics residency exposure may also be of high importance because, in some countries, maxillofacial prosthodontics is part of prosthodontics training [5]. Therefore, prosthodontics residents might receive enough exposure and training in the field to pursue an MFP career.

The results of this study can be used to attract more individuals into the profession, as factors important in the decision to purse an MFP career are described here. This survey also adds to the literature available on the practice profile of MFPs around the world. Included in the highlights of this study are the fact that some practitioners find a lack of maxillofacial prosthodontics service, and that more than 50% of respondents said that no maxillofacial prosthodontics organization exists in their country. This indicates there might be differences in need and development of maxillofacial prosthodontics services among countries.

Limitations of this study include non-randomized sampling of all individuals providing MFP services in world other than the US. A standardized recognition of MFP and availability of national MFP organizations will provide better sampling frame for this study. Due to certain practice cultures, when asked about percentage of respondents who practicing multidisciplinary treatment with other disciplines there might not be clear demarcation of individuals with Head and Neck Surgery compared to Otolaryngology.

Future studies could tap into the need, demand, and profile differences between maxillofacial prosthodontics services in resource-rich and resource-poor countries. The needs and demands for maxillofacial prosthodontics services and education around the globe are not well-documented, with the most current worldwide study dating back to the 1987 [10]. Center-specific studies were conducted in the 1986 [14] and 2001 [15]. There might be need and demand differences between more resource-rich and resource-poor countries, and cultural differences may play a role. By understanding the characteristics of a particular country, maxillofacial prosthodontics services could be maximized for that particular society. An international training criteria for MFP is needed to set the minimum standard for MFP training. Realizing the multidisciplinary nature of MFP, multidisciplinary professional organizations have the advantage to define the blueprint of such comprehensive MFP training.

The results of this study can only be extrapolated for MFPs with demographics similar to the respondents of this survey. Other limitations of this study include the variety in the number of responses to each item in the survey, as well as no detailed information available on the types of treatment provided or the reason training facilities in countries were rated unsatisfactory by respondents. Having a complete set of responses and more detailed information would provide a better picture of the population.

## Conclusions

It was found that personal satisfaction is the most important factor in the decision to choose MFP career. A university and multidisciplinary approach describe the work settings of the majority of the MFPs. There were statistically significant differences regarding the type of treatments provided by respondents with and without formal MFP training that may appeal to individuals considering pursuing this sub-specialty training.

## Abbreviations

AAMP: American academy of maxillofacial prosthetics
ACP: American college of prosthodontists
IAOFR: International academy for oral facial rehabilitation
ISMR: International society for maxillofacial rehabilitation
MFP: maxillofacial prosthodontists
